# Comment on “MRI radiomics and clinical feature model for predicting postoperative functional outcome of lumbar disc herniation: a multicenter retrospective study”

**DOI:** 10.1097/JS9.0000000000004659

**Published:** 2026-01-13

**Authors:** Zhixiao Feng, Xinran Zhu, Baoqing Zhang

**Affiliations:** a Orthopaedic Department, Second Affiliated Hospital of Tianjin University of Traditional Chinese Medicine, Tianjin, China; b Tianjin Sino-German University of Applied Sciences, Tianjin, China; c Rehabilitation Department, Second Affiliated Hospital of Tianjin University of Traditional Chinese Medicine, Tianjin, China

*Dear Editor,*

We read with great interest the article entitled “MRI radiomics and clinical feature model for predicting postoperative functional outcome of lumbar disc herniation: a multicenter retrospective study” published in your prestigious journal[1]. Existing prediction models for lumbar disc herniation (LDH) postoperative outcomes predominantly rely on subjective scoring systems and rarely integrate quantitative preoperative imaging metrics or MRI radiomics. The authors’ development of a combined clinical–radiomics model (CRM) addresses this gap and demonstrates strong predictive performance, providing a valuable tool for early outcome prediction in LDH patients. We commend their contributions, but highlight several key considerations to enhance the model’s clinical applicability and interpretability.

First, residual pain and functional impairment after LDH surgery are often linked to epidural scar adhesion and nerve compression[2]. Meanwhile, independent factors associated with failure to achieve the minimal clinically important difference (MCID) include inadequate intraoperative decompression, intraoperative nerve traction injury or iatrogenic trauma, preoperative psychological factors, and comorbidities such as the severity of diabetic neuropathy or osteoporosis^[3,4]^. Unfortunately, these key determinants were not incorporated into the current assessment of preoperative MRI muscle quality and radiomics features, which may introduce residual confounding. Although the retrospective design inevitably limits data completeness, preoperative psychological status and the severity of major comorbidities (e.g., hypertension, osteoporosis, and diabetic neuropathy) might still be feasible. Furthermore, integrating intraoperative records, early postoperative complications (e.g., infection, scarring), or immediate/follow-up postoperative MRI – ideally within a prospective framework – could improve the model’s reliability and real-world translatability.

Second, we note that in the multivariable logistic regression analysis, the 3-month postoperative PROMIS PF score was included as an independent predictor (reported with an odds ratio of approximately 1.151, *P* < 0.001). The CRM is then framed as a tool for “precise preoperative evaluation” and optimizing preoperative surgical planning. In real-world clinical practice, however, 3-month postoperative PROMIS PF data are not available at the time of preoperative decision-making. If the primary intended use is truly preoperative risk stratification, incorporating a postoperative 3-month variable raises concerns regarding temporal information leakage and feasibility. Conversely, if the authors’ actual goal is to provide a dynamic risk update at 3 months (i.e., a second-stage model predicting 6-month MCID status conditional on early postoperative recovery), this intended scenario should be explicitly clarified. Ideally, a purely preoperative version of the model should be reported alongside the dynamic model.

Third, the authors state that the CRM demonstrates strong cross-center generalizability. However, the external test cohort was formed by pooling 20 patients from Center 2 and 88 patients from Center 3 (total n = 108). While this approach increases the overall sample size, the small number of cases from Center 2 limits statistical power to evaluate center-specific performance. Moreover, all three centers belong to the same medical university system and are tertiary hospitals within the same province, suggesting that true “between-center” differences in case mix, practice patterns, or imaging infrastructure may be relatively modest. Under these circumstances, describing the CRM as having “strong cross-center generalizability” may be somewhat optimistic[5]. The supplementary results also suggest that external discrimination varies across modeling approaches; for example, the LR-based clinical–radiomics model achieved an external area under the curve (AUC) of approximately 0.69, whereas the clinical model alone achieved an AUC of approximately 0.62. More detailed reporting of cross-center calibration and a clearer discussion of potential sources of heterogeneity would further contextualize these external validation findings.

Finally, the study defined the outcome as a ≥5-point improvement in PROMIS PF at 6 months compared with baseline. While clinically intuitive, using a single dichotomous MCID threshold may reduce granularity and render model performance sensitive to the chosen cutoff. Sensitivity analyses using alternative thresholds, or complementary analyses modeling PROMIS PF change as a continuous or ordinal outcome, could provide a more comprehensive picture[6]. In addition, patients who required early revision/reoperation at the index level within 6 months were excluded. Because these cases often represent the poorest early trajectories, their systematic exclusion may narrow the clinical spectrum captured by the model[7]. Future studies could consider early reoperation as a competing or composite endpoint, or include sensitivity analyses to clarify the impact of this exclusion.

In conclusion, this multicenter retrospective study addresses an important gap in LDH outcome prediction by integrating preoperative MRI radiomics with clinical data. Targeted refinements – particularly clarifying the intended prediction time point, strengthening the interpretation of cross-center generalizability, and expanding analyses around outcome definition and early reoperation – may further enhance the clinical utility and robustness of this promising predictive tool.

We declare that the above complies with the TITAN guideline, which mandates openness in artificial intelligence reporting[8].

## Data Availability

Data availability is not applicable to this article as no new data were created or analyzed in this study.

## Declarations

Artificial intelligence (AI) tools were used only for language polishing (grammar and style). Specifically, Chatgpt 5.0 was employed on 8 December 2025, with default settings. No AI tools were used for data analysis, interpretation, or generation of scientific content. The authors confirm that they take full responsibility for the integrity and accuracy of the final manuscript.
